# Regarding “The Efficacy and Safety of Technology-Guided Dry Weight Adjustment Among Dialysis Patients: A Meta-Analysis of Randomized Controlled Trials” by Wathanavasin et al

**DOI:** 10.1016/j.xkme.2025.101204

**Published:** 2025-12-09

**Authors:** Eric P. Cohen, Bernard Canaud

**Affiliations:** 1 New York University School of Medicine, New York, New York; 2 Université de Montpellier, Montpellier, France

To the Editor,

We read with interest the comprehensive review in *Kidney Medicine* by Whathanavasin et al^1^ on the multiple technologies used for adjustment of extracellular volume in dialysis patients. Volume overload worsens morbidity and mortality of patients receiving dialysis, hence the importance of achieving euvolemia. Clinical approaches such as probing for the dry weight can significantly attenuate cardiovascular morbidity and mortality^2^ but they are subjective. Getting to euvolemia needs objective, practical, and repeatable methods.^3^ These also must genuinely reduce morbidity and mortality. Table 2 of the paper by Whathanavasin et al^1^ shows that 13 studies of such techniques yielded a combined higher number of deaths in the intervention groups (1,741 patients compared with 1,707), and more cardiovascular events in 9 studies (1,090 compared with 1,065). This difference may be a typographical transposition of the numbers. If so, then the absolute mortality benefit of the technology would be 34/1,741, a 2% benefit, and the cardiovascular event benefit 35/1,065, which is a 3% benefit. These benefits are modest, which is perhaps partly attributable to the relatively short follow-up times of the reviewed studies shown in Table 1, which averaged only 14 months.^1^ Pending longer-duration studies from well-designed trials of technological guidance, such as bioimpedance, we advise that clinical probing for dry weight remains the standard of care in the management of patients receiving hemodialysis, that is, the achievement of euvolemia as guided by symptoms, physical examination, and blood pressure.^2^
